# Anthromes displaying evidence of weekly cycles in active fire data cover 70% of the global land surface

**DOI:** 10.1038/s41598-019-47678-4

**Published:** 2019-08-06

**Authors:** J. M. C. Pereira, M. A. Amaral Turkman, K. F. Turkman, D. Oom

**Affiliations:** 1 Centro de Estudos Florestais, Instituto Superior de Agronomia, Universidade de Lisboa, Lisboa, Portugal; 2 Centro de Estatística e Aplicações, Faculdade de Ciências da Universidade de Lisbon, Lisboa, Portugal

**Keywords:** Environmental impact, Natural hazards

## Abstract

Across the globe, human activities have been gaining importance relatively to climate and ecology as the main controls on fire regimes and consequently human activity became an important driver of the frequency, extent and intensity of vegetation burning worldwide. Our objective in the present study is to look for weekly cycles in vegetation fire activity at global scale as evidence of human agency, relying on the original MODIS active fire detections at 1 km spatial resolution (MCD14ML) and using novel statistical methodologies to detect significant periodicities in time series data. We tested the hypotheses that global fire activity displays weekly cycles and that the weekday with the fewest fires is Sunday. We also assessed the effect of land use and land cover on weekly fire cycle significance by testing those hypotheses separately for the Villages, Settlements, Croplands, Rangelands, Seminatural, and Wildlands anthromes. Based on a preliminary data analysis of the daily global active fire counts periodogram, we developed an harmonic regression model for the mean function of daily fire activity and assumed a linear model for the de-seasonalized time series. For inference purposes, we used a Bayesian methodology and constructed a simultaneous 95% credible band for the mean function. The hypothesis of a Sunday weekly minimum was directly investigated by computing the probabilities that the mean functions of every weekday (Monday to Saturday) are inside the credible band corresponding to mean Sunday fire activity. Since these probabilities are small, there is statistical evidence of significantly fewer fires on Sunday than on the other days of the week. Cropland, rangeland, and seminatural anthromes, which cover 70% of the global land area and account for 94% of the active fires analysed, display weekly cycles in fire activity. Due to lower land management intensity and less strict control over fire size and duration, weekly cycles in Rangelands and Seminatural anthromes, which jointly account for 53.46% of all fires, although statistically significant are weaker than those detected in Croplands.

## Introduction

Across the globe, human activities have been gaining importance relatively to climate and ecology as the main controls on fire regimes, and during the last few decades anthropogenic vegetation burning expanded its impact on land cover, ecosystem dynamics, and atmospheric greenhouse gas emissions from regional to global scales^1–4^. Human activity became a significant driver of the frequency, extent and intensity of vegetation burning worldwide, and the timing of ignitions is strongly dependent on human agency^5,6^. Fire temporal patterns are a function of climate dynamics and human activity, with periodic cycles found at annual scale in seasonally dry areas, and at the daily scale in response to underlying cycles in meteorological variables and land management activities^6,7^. Fire regimes have been modified to manage croplands, rangelands, forests, and wildlife, to protect infrastructure and settled areas^8^, as a form of political protest^9^, and to wage war^10^. Predictability of spatial and temporal fire patterns increases with the length of human presence and the degree of human dominance over the landscape^11^. Vegetation burning has been analyzed at time scales ranging from daily^7^ to annual^12^, centennial^13^, and millennial^14,15^. It is a large source of greenhouse gases and aerosols and constitutes a major factor controlling variability of atmospheric composition at the inter-annual scale^16^.

Approaches to isolate the impact of anthropogenic aerosol on clouds from natural cloud variability were reviewed by^17^ and included the detection of weekly cycles, because seven-day periodicities are highly unlikely due to natural forcing, and thus provide strong evidence of anthropogenic perturbations. Weekly cycles over broad geographical areas cannot be explained by the reduction in industrial and transportation emissions in urban areas alone. The possible anthropogenic influence on these cycles remains unclear, but different processes are required to explain their occurrence. The most plausible hypothesis to interpret large-scale weekly cycles is through direct and indirect effects of anthropogenic aerosols, whose emissions also tend to show weekly periodicity at large-scale^18^. A weekly cycle of aerosol optical depth (AOD), observed in the United States and in Central Europe, was stronger in urban sites than in rural sites. A different weekly cycle detected in the Middle East and India had lower AODs on Thursday and Friday, the weekend for Middle Eastern cultures^19^. Close synchrony between weekly peaks in anthropogenic aerosols and tornado activity over the eastern US during the summer was detected by^20^. They found a statistically significant weekly cycle in storm activity during summer for the period 1995 to 2009, unlikely to be due to natural variability. A connection with fire activity was provided by^21^ in their analysis of an historical tornado outbreak in the southeastern U.S., where they proposed that smoke from biomass burning in Central America might have increased tornado severity due to boundary-layer stabilization by soot and an increase in the optical thickness of lower tropospheric clouds. The assumption that vegetation burning is a natural aerosol source, and thus does not display weekly cycles, was adopted in the analyses of^20^ and^21^, as well as in the atmospheric chemistry modelling studies of^22^ and^17^. However,^23^ found significantly weekly cycles in sub-Saharan Africa cropland burning, with Sunday minima in predominantly Christian regions and Friday minima in mainly Muslim regions. They did not find significant weekly cycles in fire activity in densely settled areas, where vegetation burning is very limited, nor in rangelands, forests and wildlands, where fire management intensity is weaker than in croplands.

These continental-scale findings, combined with the recognition of the strongly anthropogenic nature of global vegetation burning^24^ motivated the hypothesis of weekly cycles in global fire activity, as observed with remotely sensed active fire data. The hypothesis of a Sunday weekly minimum in fire activity has two justifications: (i) international labour legislation stipulates a weekly day of rest, which in most countries is Sunday, and (ii) of the two major global religions (Christianity and Islam) that prescribe a weekly day of rest, Christianity prevails in more high fire incidence areas. We also assessed the effect of land use and land cover on weekly fire cycle significance by testing the aforementioned hypotheses separately for the Villages, Settlements, Croplands, Rangelands, Seminatural, and Wildlands anthropogenic biomes (anthromes)^25^, which map global patterns of sustained direct human interaction with ecosystems. Anthrome-specific analysis provides valuable information to elucidate the land management activities, regions, and times of the year contributing to the occurrence of weekly cycles in global vegetation burning.

**Figure 1:**
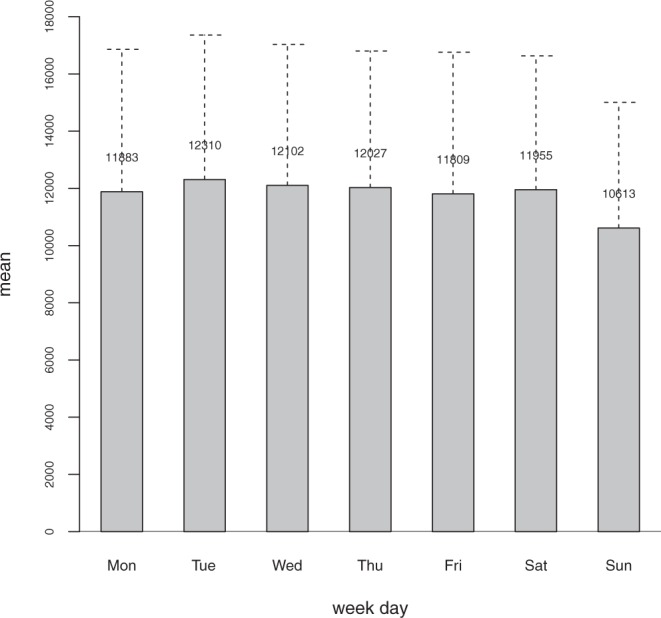
Mean + 1.s.d. active fire counts per weekday for the entire study period. Tuesday, Wednesday and Thursday are the weekdays with the highest fire count averages, exceeding 12,000; Monday, Friday and Saturday all exceed 11,000 counts, and Sunday is the only day with a fire count average below 11,000.

**Figure 2:**
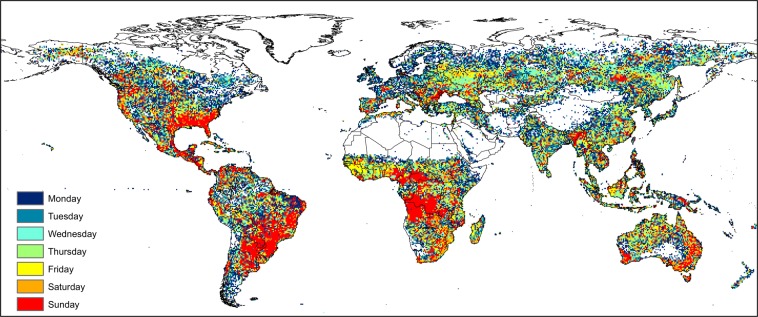
Weekday with the fewest fire counts, 2002–2012.

Previous research by^26^ and^27^ also detected Sunday minima in global fire activity, using spatially aggregated fire counts data from the NASA Earth Observations website (http://neo.sci.gsfc.nasa.gov/view.php?datasetId=MOD14A1_M_FIRE). However, inconsistencies between the active fire numbers reportedly used by^26^ and by^27^, and their discrepancies relatively to other published sources justify revisiting the topic of weekly cycles in global active fire data. Their data differ from those published by various other sources in the number of MODIS active fires reportedly used, in the time series of annual active fire numbers, and in the weekly distribution of active fires. In comparison with all other published sources that have partially overlapping study periods and mention the numbers of active fires used,^26^ claim having worked with many more fires, while^27^, who used a time series three years longer than that of^26^, surprisingly report substantially fewer fires. Table 1 in Supplementary Information shows the numbers involved in these comparisons. The discrepancies between^26^ and^27^ relatively to other authors are largest for the first two years of their time series, 2001 and 2002, which they claim to have the most active fires. This is an obvious impossibility, since during all of 2001 and the first half of 2002, only the TERRA instrument, which has a morning overpass at 10:30AM/PM, was in orbit. AQUA was launched in mid-2002 with a 2:30AM/PM overpass and that more than doubled the amount of active fires detected because the AQUA daytime overpass observes the most active period of the diurnal cycle of vegetation burning. Figure 5 of^28^ displays the relative contribution of AQUA to the total MODIS active fire detections and clearly shows that AQUA detections account for more than half of all active fires. Therefore, the number of active fires in the MODIS data product (MCD14ML) for the years 2001 and 2002 is less than half of that in each subsequent year, as shown in figure 2a of^29^. Table 1 in Supplementary Information provides specific comparisons for these two years, taking into account differences in the MODIS sensors and in the active fire data collections used by the various authors. There are also differences between the weekly distributions of MODIS active fire counts shown in figure 2a of^26^ and in figure 4 of^27^, and the distribution portrayed in Fig. 1 below. In the original MDC14ML data that we used the relative difference between the numbers of active fires on Sunday and Monday is larger, i.e., Sunday is a more prominent weekly minimum and Tuesday is a more prominent weekly maximum, and thus the magnitude of the weekly cycle in the number of active fires is larger. In addition, there is a change in rank order of the number of active fires by weekday with Friday, rather than Monday, having the second fewest fires. These differences are likely to affect the results of a weekly cycle analysis.

The paper by^26^ also has some methodological issues. In our opinion, the statistical tests they used to detect and characterize weekly fire cycles, may not be adequate to answer two fundamental questions: (i) if there is evidence in the data that there are 7 day cycles (ii) If there is such evidence in the data, fire activity in each day of the week have different statistical properties, in particular, if average Sunday fire counts are less that the average counts in other days of the week. Daily counts of fire activity constitute a time series and by nature, they show strong and complex temporal dependence structures. Without accounting for such temporal dependence structures it is not possible to come up with correct conclusions on (i) and (ii). The authors^26^ base their results on statistical methods, which we believe, do not take into account proper time series analysis. Although their Monte Carlo tests may show the extent to which a weekly cycle is present under random conditions, and thus the probability that the weekly pattern of fire counts observed in the data occurs by chance, they do not test the shape, nor even the existence of such a cycle. Their subsequent Kolmogorov-Smirnov test only shows, for the global scale analysis, that the two days of the week displaying the largest and smallest number of fires (Tuesday and Sunday respectively) have distinct statistical distributions. This, by itself, does not provide evidence that there is a minimum on Sunday fire activity, neither shows that the activity on Sunday differs from the other days of the week.

Our goal in the present study is to reanalyse the topic of weekly cycles in vegetation fire activity at global scale, relying on the original MODIS active fire detections at 1 km spatial resolution (MCD14ML) with appropriate statistical methodologies to detect significant periodicities in time series data. Using Bayesian methodology, a simultaneous 95% credible band for the mean function was constructed. Sunday minimum is hence directly investigated by computing the probabilities that the mean functions of every week day (Monday to Saturday) are inside the credible band corresponding to mean Sunday fire activity.

## Data

Daily fire activity data were obtained from the NASA MODIS MCD14ML Collection 5 active fire product^30^ at 1 km spatial resolution, after screening for false alarms and non-vegetation fires according to the procedures detailed in^29^. We analysed the period from July 8, 2002 to July 29, 2012 (TERRA and AQUA sensors), for a total of 525 full weeks and 43,416,629 fire counts. Figure 1 shows the mean + 1 sd. of total fire counts per weekday. Land use/land cover data used are from the Anthropogenic Biomes of the World (anthromes) dataset, at 5′ (ca. 9 km) spatial resolution, dated from the year 2000. Anthrome data combine information on population density, land cover and land use to derive a map of global patterns of sustained direct human interaction with ecosystems^25^. Since a large majority of global vegetation burning is anthropogenic^24^, they were considered particularly appropriate for our analysis. The relative stability of spatial patterns depicted by the anthromes dataset further justifies its use in conjunction with global fire activity data for the period 2002 to 2012.

**Figure 3:**
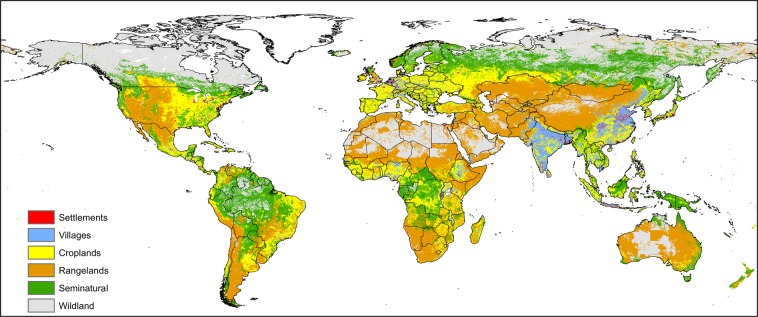
Global Anthromes (v2.0) aggregated at 0.5° spatial resolution using the modal class rule.

Each active fire was labeled with the anthrome class present at its geographical coordinates to create the datasets for the anthrome-specific fire weekly cycle analyses (see Table 1). Maps aggregated at 0.5° spatial resolution of the weekday with the fewest fires and of Global Anthromes (v2.0) using the modal class rule are shown in Figs 2 and 3 respectively.

**Table 1:** Number and percent distribution of active fires by anthrome (July 2002–July 2012).

Anthromes	Settlements	Villages	Croplands	Rangelands	Seminatural	Wildlands
fires	139420	1545653	17043106	9111319	13555751	1006472
%	0.33	3.65	40.19	21.49	31.97	2.37

## Methods and Models

### Preliminary data analysis and sampling model identification

To search for weekly cycles in vegetation burning, we analysed a 525-week time series of global daily MODIS active fire counts.

The sample auto correlation (acf) and the partial auto correlation function (pcf) together with the periodogram of the log-transformed daily global fire count data were used to search for periodical variations in the data. The periodogram was calculated to identify frequencies at which jumps occur and their significance was tested with Fisher’s test and Whittle’s test^31,32^.

**Figure 4:**
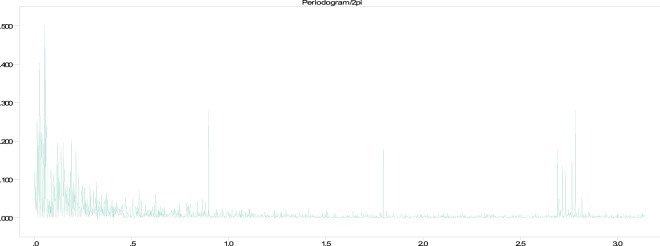
The periodogram of log transformed time series of global counts upon deseasonalizing the fundamental frequency and it first 3 harmonics.

The periodogram showed statistically significant jumps at the fundamental frequency *w*_*j*_ = 2*πj*/365 corresponding to 1 cycle in 365 days and its first 3 harmonics, corresponding respectively to 1 cycle in 6 months, 4 months, and 3 months. De-seasonalizing the data by fitting an harmonic regression at these frequencies unmasked further jumps in the periodogram at frequencies corresponding respectively to 1 cycle in 7 days and 1 cycle in 3 and half days. Further application of Whittle’s test showed that these two jumps are also statistically significant. Figure 4, shows the periodogram of the deseasonalized series where these jumps are quite evident. Jumps on the right hand side of the periodogram correspond to high frequency noise.

Results of the spectral analysis clearly suggest that the log transformed global daily fire count time series *Y*_*t*_ is non-stationary, and that its mean function *M*_*t*_ should include a harmonic sum with k = 6 harmonics corresponding to the angular frequencies of 1 cycle per year, per 6, 4, and 3 months, and per 7, and 3.5 days. We then suggest the model *Y*_*t*_ = *M*_*t*_ + *X*_*t*_, with *X*_*t*_ a stationary error term. Detailed study of the deseasonalized series by using the software ITSM^31^ suggested that the stationary component of the time series *X*_*t*_ is well represented by a zero mean stationary and invertible Gaussian Autoregressive model of order 7 (AR(7)).

The annual and multi-month cycles in fire activity result from global climate seasonality patterns, including the lagged dry seasons of the Northern and Southern hemispheres, as well as the occurrence of fire seasons in the tropical savannas of the Northern hemisphere during the boreal winter^6,33^. We hypothesize that the weekly cycle is an anthropogenic fingerprint, due to weekly days of rest determined by religious norms and labor regulations. Figure 1 shows that there are four consecutive weekdays with a mean number of fires under 12,000 (Friday to Monday), followed by three days with over 12,000 fire counts (Tuesday to Thursday). Since Friday is a religious holiday for Muslims in the extensively burned sub-Saharan region of Northern hemisphere Africa, Saturday is a civil day of rest, and Sunday is simultaneously a civil and religious holiday in most of the western world, the reduced fire activity during these days is understandable. The inclusion of Monday in this group of days may be a spillover effect from the previous days. The period Tuesday to Thursday is not affected by imposed weekly days of rest and thus the use of fire as a land management tool increases.

### Bayesian model and inference

Following the preliminary data analysis we assumed the following model for the time series of log-transformed daily global fire count data

1 $${Y}_{t}={M}_{t}+{X}_{t},$$

where the mean function *M*_*t*_ is

2 $${M}_{t}={a}_{0}+{a}_{1}t+\mathop{\sum }\limits_{i=1}^{6}\,[{c}_{i}\,\cos \,{\omega }_{i}t+{d}_{i}\,\sin \,{\omega }_{i}t],$$

with angular frequencies *ω*_*i*_, *i* = 1,2, .. 6 corresponding to the significant jumps identified in the periodogram and

$${X}_{t}=\mathop{\sum }\limits_{i=1}^{7}\,{\varphi }_{i}{X}_{t-i}+{Z}_{t}$$

is a stationary, zero mean process compatible with a stationary and invertible Gaussian *AR*(7) process, where, *Z*_*t*_ is a zero mean, uncorrelated (white noise) process.

For the vector of parameters of the fixed effects (*a*_1_, *c*_*i*_, *d*_*i*_, *i* = 1, ..., 6) we considered independent normal priors with zero mean and precision 0.001 and a flat prior for *a*_0_. For the parameters of the AR(7) process, (*ϕ*_1_, ..., *ϕ*_7_) we used, after a transformation of the parameter space, penalised complexity priors for the partial autocorrelation functions, as suggested by^34^.

We fitted this model within the R-INLA environment^35^, in which the mean function *M*_*t*_ and the stationary process *X*_*t*_ appear respectively as the fixed and random effects in the linear model specification.

From the analysis of the model, we observe from Table 2 that all the harmonic coefficients are significant, but there is no evidence of a linear trend.

**Table 2:** Summary Statistics for the posterior distribution of the fixed effects.

	mean	sd	0.025quant	0.5quant	0.975quant	mode
*a*_0_	9.3405E+00	2.8958E-02	9.2834E+00	9.3405E+00	9.3974E+00	9.3405E+00
*a*_1_	−2.3729E-05	1.3648E-05	−5.0555E-05	−2.3743E-05	3.1441E-06	−2.3770E-05
*c*_1_	1.8654E-01	2.0232E-02	1.4674E-01	1.8653E-01	2.2634E-01	1.8652E-01
*d*_1_	2.6376E-01	2.0361E-02	2.2370E-01	2.6376E-01	3.0381E-01	2.6375E-01
*c*_2_	1.7998E-01	1.9686E-02	1.4126E-01	1.7998E-01	2.1869E-01	1.7997E-01
*d*_2_	1.1867E-01	1.9764E-02	7.9798E-02	1.1866E-01	1.5753E-01	1.1864E-01
*c*_3_	−4.7871E-02	1.8884E-02	−8.5001E-02	−4.7875E-02	−1.0761E-02	−4.7881E-02
*d*_3_	1.3137E-01	1.8947E-02	9.4127E-02	1.3136E-01	1.6861E-01	1.3135E-01
*c*_4_	−6.7088E-03	1.7937E-02	−4.1964E-02	−6.7110E-03	2.8522E-02	−6.7141E-03
*d*_4_	−8.6432E-02	1.7989E-02	−1.2178E-01	−8.6438E-02	−5.1089E-02	−8.6449E-02
*c*_5_	−4.2395E-02	4.9663E-03	−5.2150E-02	−4.2395E-02	−3.2648E-02	−4.2396E-02
*d*_5_	1.0734E-02	4.9673E-03	9.7628E-04	1.0734E-02	2.0483E-02	1.0734E-02
*c*_6_	−3.3660E-02	2.8948E-03	−3.9344E-02	−3.3660E-02	−2.7980E-02	−3.3660E-02
*d*_6_	−1.0493E-02	2.8947E-03	−1.6177E-02	−1.0493E-02	−4.8134E-03	−1.0493E-02

### Comparing the mean functions

Once we establish that fire activity indeed displays weekly cycles, it is interesting to see in which day of the week the minimum fire activity is observed, in particular, one may want to test the hypothesis that the fire activity is minimum on Sundays. One way of answering this question is to compare the mean functions ${M}_{{t}_{i}}$ in (2) with *t*_*i*_ = *i*, *i* + 7, *i* + 14, ... and *i* = 1, ..., 7, where *i* = 1 refers to Monday and *i* = 7 refers to Sunday. That is compare the mean functions corresponding to the fire activity series of different days of the week.

Our strategy to test the hypothesis that the fire activity is minimum on Sundays will be based on calculating the 95% simultaneous credible interval (credible band) corresponding to Sunday mean function ${M}_{{t}_{7}}$ and (i) check how likely is the mean function of other days of the week falling within this credible band. Additionally we compute the probabilities that the mean functions of other days of the week are above the estimated mean function of Sunday (ii). Significant lower probabilities of (i) and significant higher probabilities of (ii) give evidence that indeed fire activity is minimum on Sundays. We explain below the computational details.

The marginal posterior distribution for the mean function *M*_*t*_, *t* = 1, ..., 3765 is approximately multivariate normal with posterior mean ${\hat{M}}_{t},t=1,\ldots ,3765$ and posterior covariance matrix *S*^35^, which can be obtained from R-INLA using inla.make.lincomb function. Pointwise 100(1 − *γ*)% HPD (highest posterior density) intervals for each component of the vector *M*_*t*_ can also de obtained using the function inla.hpdmarginal. However, rather than obtaining pointwise credible intervals, one should seek for a simultaneous credible band for *M*_*t*_ of size 100(1 − *α*)%. For that we follow the suggestion given by^36^.

For the random vector {*M*_*t*_, *t* = 1, ..., *n*} = (*M*_1_, ..., *M*_*n*_), a 100(1 − *α*)% simultaneous credible band can be defined as a hyper-rectangular region

$$R=\{\{{M}_{t}\}:{\cap }_{i=1}^{n}({M}_{i}\in {I}_{i,\gamma })\}$$

where {*I*_*i*,*γ*_}, *i* = 1, ..., *n* denotes a set of individual 100(1 − *γ*)% HPD credible intervals for each component of the vector *M*_*t*_, with *γ* chosen so that *P*({*M*_*t*_} ∈ *R*) = 1 − *α*.

Since the posterior distribution for the vector *M*_*t*_ is approximately multivariate normal with known mean vector and known covariance matrix, by trial and error, one can obtain a simultaneous 100(1 − *α*)% HPD credible band for the vector {*M*_*t*_, *t* = 1, ..., *n*}, by computing, for every individual component of the vector, a 100(1 − *γ*)% HPD credible interval, varying *γ* till the overall probability is 1 − *α*.

$$P(\{{M}_{t},t=1,\ldots ,n\}\in R)=1-\alpha .$$

Actually this can also be obtained using the function excursions.simconf available in the R package excursions^37^.

Considering the fact that the linear trend was not significant and considering the cyclic nature of *M*_*t*_, above calculations were reported for the first 52 weeks, rather than for the full period.

**Figure 5:**
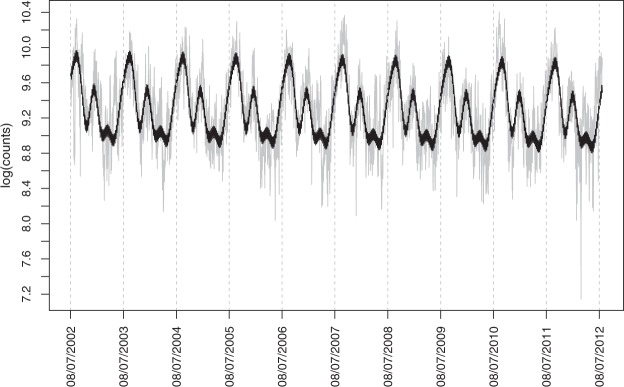
Logarithm of the daily fire counts (in grey) and the fitted mean function (in black).

**Figure 6:**
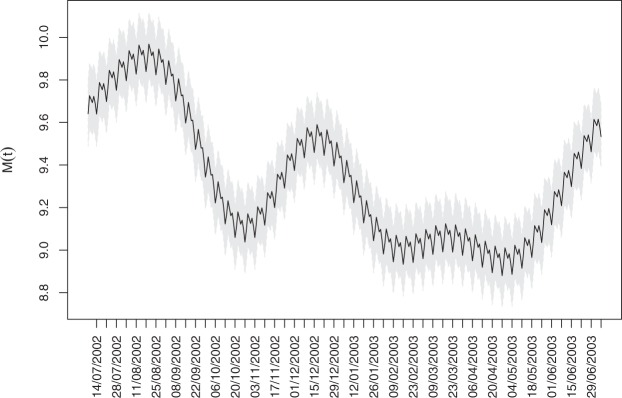
Estimated posterior mean function ${\hat{M}}_{t},t=1,\ldots ,364$ together with the 95% simultaneous credible bands (in grey).

In Figure 5 we display the series of the log counts with the estimated mean function (posterior mean) ${\hat{M}}_{t}$, and in Fig. 6 the estimated mean function for the first year, ie, ${\hat{M}}_{t},t=1,\ldots ,364$ together with the 95% simultaneous credible band.

Since the posterior distribution of the mean function *M*_*t*_ is multivariate normal, *M*_*t*_ can be partitioned into ${M}_{{t}_{i}},i=1,\mathrm{...},7$ (with 1 for Monday, 7 for Sunday), as explained before, to represent the mean functions for the different days of the week. Properties of the multivariate normal distribution imply that the posterior distributions of ${M}_{{t}_{i}}$, *t*_*i*_ = *i*, *i* + 7, ... and *i* = 1, ..., 7 are also approximately multivariate normal with means given by the ${\hat{M}}_{{t}_{i}}$, *t*_*i*_ = *i*, *i* + 7, ... and *i* = 1, ..., 7 and corresponding covariance matrices, which are sub-matrices of the overall covariance matrix. Also if we represent the 100(1 − *α*)% HPD credible bands for *M*_*t*_ by the two series *LB*_*t*_ and *UP*_*t*_, they can similarly be partitioned into $L{B}_{{t}_{i}}$ and $U{P}_{{t}_{i}}$, for *i* = 1, ..., 7.

We propose as a methodology to test the hypothesis that there is, on average, smaller fire activity on Sundays, by computing the probabilities, for *i* = 1, ..., 6

$$P(L{B}_{{t}_{7}}\le {M}_{{t}_{i}}\le U{B}_{{t}_{7}}),$$

that is, the probabilities that the mean function for every day of the week is inside the credible bands corresponding to Sunday, ie, inside $L{B}_{{t}_{7}}$ and $U{P}_{{t}_{7}}$. These probabilities can be easily obtained using the function gaussint() from the R package excursions^37^.

In Table 3 we show these probabilities, with the associated error in brackets, computed using the first year. For the other years the results are very similar.

**Table 3:** Probabilities that mean functions for the logarithm of the fire counts for each day of the week are inside the 95% simultaneous credible band for Sunday.

Monday	Tuesday	Wednesday	Thursday	Friday	Saturday	Sunday
0.1965 (0.0036)	0.0066 (0.0006)	0.0712 (0.0022)	0.3140 (0.0041)	0.2041 (0.0033)	0.7019 (0.0039)	0.9696 (0.0013)

We also compute the posterior probabilities that the mean function for every other week day ${M}_{{t}_{i}},i=1,\mathrm{...},7$ is above the estimated mean function for Sunday (the mean of the posterior distribution of the mean vector ${M}_{{t}_{7}}$). These probabilities are given in Table 4.

**Table 4:** Probabilities $P=P({M}_{{t}_{i}} > {\hat{M}}_{{t}_{7}})$ that mean functions for the logarithm of the fire counts for each day of the week are above the estimated mean function for Sunday.

	Monday	Tuesday	Wednesday	Thursday	Friday	Saturday	Sunday
P	0.0556 (0.0012)	0.7644 (0.0033)	0.6303 (0.0037)	0.4531 (0.0037)	0.7412 (0.0034)	0.3511 (0.0036)	0.0172 (0.0007)

From Table 3 we observe that, besides Saturday, the mean functions of all the other days of the week have lower probabilities of being inside the credible band for Sunday. Also from Table 4 we observe that except for Monday, all other days of the week have relatively high probabilities of being above the estimated mean function of Sunday. Hence there is some evidence that, in general, minimum fire activity is indeed on Sundays.

**Figure 7:**
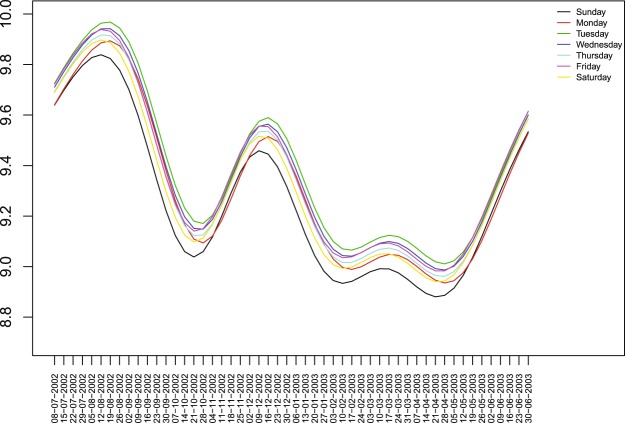
Estimated mean functions ${\hat{M}}_{{t}_{i}}$ of the logarithm of the global fire counts for each day of the week.

Figure 7 is an unfolded reproduction of Fig. 6, that is, the estimated mean function ${\hat{M}}_{t}$ is partitioned into ${\hat{M}}_{{t}_{i}}$, *t*_*i*_ = *i*, *i* + 7, *i* + 14, ..., and *i* = 1, ..., 7 to represent the estimated mean function for the different days of the week. For ease of reading, only the first year is plotted. Other years have the same behaviour, due to the cyclic nature of the mean function, since the linear trend is not statistically significant.

**Figure 8:**
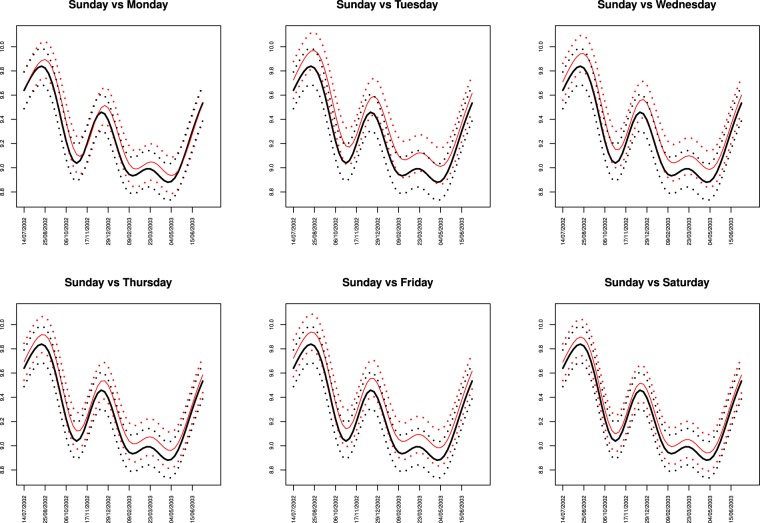
Estimated mean function of the logarithm of the fire counts for Sunday(in black) and corresponding 95% simultaneous credible band (black dots), with the estimated mean functions ${\hat{M}}_{{t}_{i}}$ of the logarithm of the fire counts for each other day of the week(in red) and corresponding simultaneous credible band(red dots).

It is clear from that plot that the estimated mean function corresponding to Sunday fire counts is smaller than the other estimated mean functions almost uniformly for every week. Looking in more detail we remark that the estimated mean function for Sunday shows always lower values, except for the period ranging from the second week of November to the first week of December and from the third week of May until the end of June, when it is always very close to the estimated mean function for Monday. The second minimum is basically on Saturday or Monday. This may indicate the existence of a weekend effect on daily fire counts. To understand how much smaller the estimated mean function for Sunday is from the other days of the week, we display in Fig. 8 the estimated mean function for Sunday together with the corresponding 95% simultaneous credible band and the estimated mean functions for each of the other days of the week. We observe that the estimated mean function for Saturday is always inside the credible band for Sunday and only during two periods of the year, namely, third week of August to third week of October and second week of December to the second week of April, the estimated mean functions for some of other days of the week are above the upper bound of the credible band for Sunday. (see detailed study in the technical report)^38^ (http://ceaul.org/wp-content/uploads/2018/10/NotaseCom_3_2016.pdf). This is an indication that there is evidence of a weekly cycle with minimum on Sunday, only during these periods of the year.

Further, the comparison of fire activity in each day of the week involves comparison and ordering of time series, We note that existence of ordering for mean functions does not necessarily imply stochastic ordering for the respective time series. However the following result in^39^ gives an indication when ordering in mean functions imply respective stochastic ordering: If *X*_*t*_ and *Y*_*t*_ are two independent Gaussian processes with the same covariance structure and if *E*(*X*_*t*_) ≤ *E*(*Y*_*t*_) uniformly for every *t*, then *X*_*t*_ is stochastically dominated by *Y*_*t*_. The precise definition of stochastic ordering can be found in^39^. There is strong evidence in the data that deseasonalized log fire activity for each day of the week follow a AR(1) process and although they are certainly not independent, this might suggest that the results on comparison on the means may extend to respective stochastic ordering. However, further detailed work is needed to reach such complete conclusion.

## Anthrome-level Analysis

**Figure 9:**
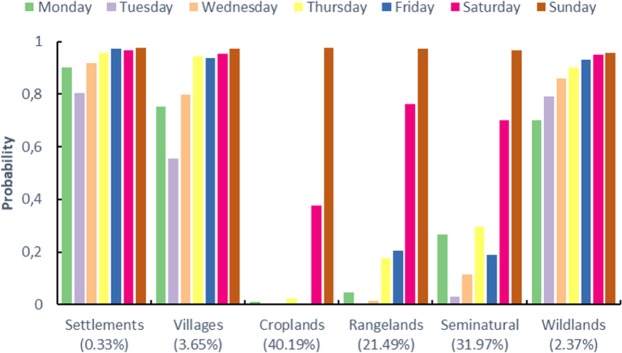
Anthrome-specific probabilities that mean functions for the logarithm of the fire counts for each day of the week are within the 95% simultaneous credible band for Sunday: complementary to Table 5. Values in parenthesis represent the % of total active fires in the respective anthrome.

The methodology shown in section 3.3 was applied to time series of the logarithm of the number of active fire counts described with each of the six anthromes shown in Fig. 3. Following their global analysis,^26^, searched for weekly cycles in regions of the globe displaying abundant fire activity. This approach designated^40^ post-hoc domain selection, and warn that it risks biasing the analysis, since it might be expected that regions experiencing many fires might exhibit large weekly cycles. In order to avoid the potential for this type of bias, we regionalised our analysis independently of any observed fire activity spatial patterns, based on the hypothesis that the likelihood of active fire weekly cycles vary by anthrome. Only for Croplands did Whitle’s test show strong evidence of significant jumps in the periodogram at frequency corresponding to weekly cycles, although some evidence is also observed for Rangelands and Seminatural anthromes. Table 5 and Fig. 9 show, for each anthrome, the probabilities that the mean function for every day of the week is inside the Sunday 95% credible band.

**Table 5:** Anthrome-specific probabilities that mean functions for the logarithm of the fire counts for each day of the week are within the 95% simultaneous credible band for Sunday (error in brackets).

Anthrome	Monday	Tuesday	Wednesday	Thursday	Friday	Saturday	Sunday
Croplands	0.0122 (0.0007)	0.0000 (0.0000)	0.0002 (0.0000)	0.0227 (0.0012)	0.0013 (0.0002)	0.3770 (0.0040)	0.9750 (0.0012)
Rangeland	0.0455 (0.0017)	0.0005 (0.0002)	0.0145 (0.0009)	0.1779 (0.0032)	0.2051 (0.0033)	0.7628 (0.0037)	0.9724 (0.0013)
Seminatural	0.2684 (0.0041)	0.0309 (0.0014)	0.1153 (0.0027)	0.2946 (0.0040)	0.1890 (0.0033)	0.6995 (0.0039)	0.9673 (0.0014)
Wildland	0.7001 (0.0043)	0.7898 (0.0037)	0.8584 (0.0032)	0.9026 (0.0027)	0.9293 (0.0023)	0.9487 (0.0020)	0.9554 (0.0020)
Settlements	0.9011 (0.0027)	0.8047 (0.0035)	0.9164 (0.0024)	0.9566 (0.0018)	0.9729 (0.0014)	0.9653 (0.0015)	0.9770 (0.0012)
Villages	0.7517 (0.0039)	0.5565 (0.0045)	0.7974 (0.0036)	0.9440 (0.0020)	0.9384 (0.0021)	0.9526 (0.0019)	0.9742 (0.0013)

**Figure 10:**
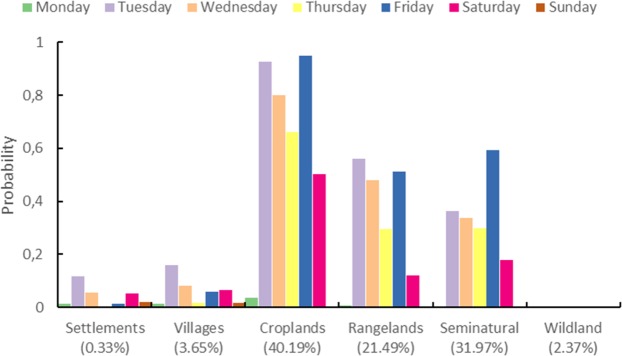
Probabilities that the mean function for every day of the week is above the estimated mean function for Sunday: complementary to Table 6. Values in parenthesis represent the % of total active fires in the respective anthrome.

Complementary to these probabilities, we also calculated the probability that a mean function of a day of the week is above the estimated Sunday mean function. These probabilities are given in Table 6 (see also Fig. 10).

**Table 6:** Probabilities that the mean function for every day of the week is above the estimated mean function for Sunday (error in brackets).

Anthrome	Monday	Tuesday	Wednesday	Thursday	Friday	Saturday	Sunday
Croplands	0.0367 (0.0012)	0.9273 (0.0022)	0.8004 (0.0032)	0.6606 (0.0038)	0.9492 (0.0017)	0.5014 (0.0039)	0.0010 (0.0001)
Rangeland	0.0069 (0.0003)	0.5622 (0.0038)	0.4803 (0.0037)	0.2961 (0.0033)	0.5133 (0.0038)	0.1209 (0.0022)	0.0014 (0.0002)
Seminatural	0.0034 (0.0003)	0.3630 (0.0038)	0.3361 (0.0037)	0.2981 (0.0035)	0.5919 (0.0040)	0.1801 (0.0029)	0.0013 (0.0002)
Wildland	0.0001 (0.0000)	0.0002 (0.0000)	0.0002 (0.0001)	0.0005 (0.0001)	0.0010 (0.0001)	0.0008 (0.0002)	0.0012 (0.0001)
Settlements	0.0142 (0.0008)	0.1173 (0.0025)	0.0560 (0.0017)	0.0041 (0.0004)	0.0146 (0.0007)	0.0522 (0.0015)	0.0199 (0.0008)
Villages	0.0150 (0.0006)	0.1597 (0.0027)	0.0821 (0.0019)	0.0172 (0.0007)	0.0588 (0.0015)	0.0664 (0.0017)	0.0167 (0.0007)

Comparing the probabilities between Sunday and other weekdays displayed on Tables 5 and 6 we can say that there is a strong evidence that for Croplands, the mean function for Sunday is statistically smaller than the mean function for every other day of the week. This evidence is not so strong for Rangelands and Seminatural since the probability that Saturday mean function is inside the 95% credible band for Sunday is relatively high. For other anthromes there is no statistical evidence of a difference between the mean functions corresponding to the different days of the week. Globally, albeit with varied strengths of evidence, three anthromes (croplands, rangelands, and seminatural areas), corresponding to 70% of the land surface and to 94% of the active fire data analysed display weekly cycles in fire activity.

**Figure 11:**
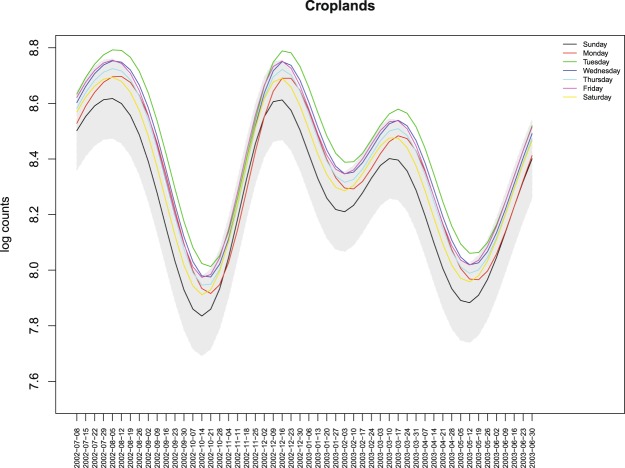
Estimated mean function of the logarithm of active fire counts in croplands for Sunday and corresponding 95% simultaneous credible band(in grey), with the estimated mean functions ${\hat{M}}_{{t}_{i}}$ of the logarithm of the fire counts for every other day of the week.

To understand how much smaller the estimated mean function for Sunday in Croplands is from the other days of the week, we display in Fig. 11 the estimated mean function for Sunday together with the 95% simultaneous credible band and the estimated mean functions for each of the other days of the week.

**Figure 12:**
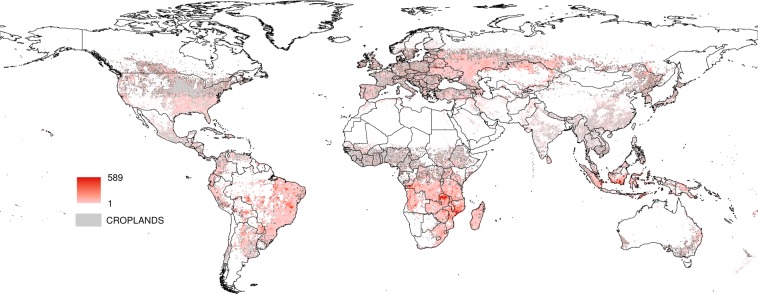
Number of active fires per 0.5° grid cell between early September and early October, when the weekly cycle is strongest in the cropland anthrome.

**Figure 13:**
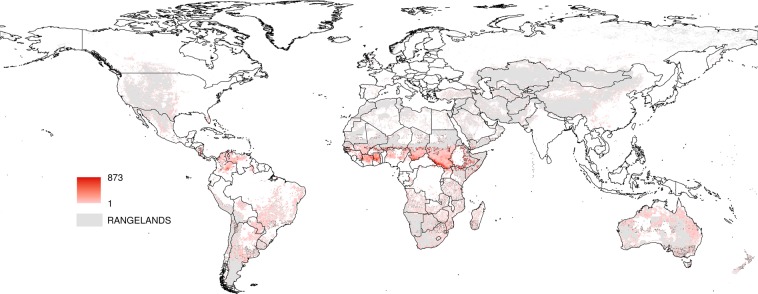
Number of active fires per 0.5° grid cell between late December and late January, when the weekly cycle is strongest in the rangeland anthrome.

**Figure 14:**
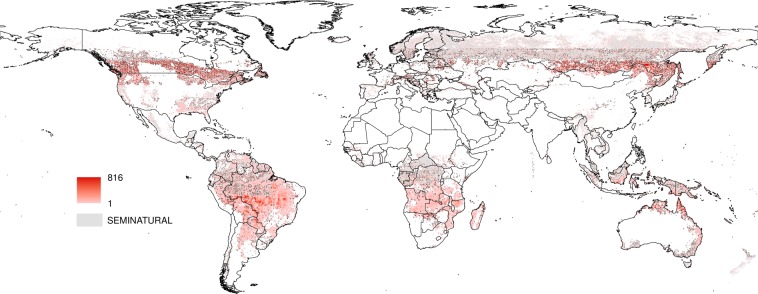
Number of active fires per 0.5° grid cell between mid-September and early October, when the weekly cycle is strongest in the seminatural anthrome.

For each anthrome, we identified the longest uninterrupted period during which the estimated mean function for the largest number of weekdays is above the 95% upper credible band for Sunday, representing the time of the year when the weekly cycle of fire activity is strongest. In the Cropland anthrome this period lasts from early September to early October, when vegetation burning takes place mostly in Southern hemisphere Africa, Brazil, Southeastern USA, Southwestern Russia and the Ukraine (see Fig. 12). The Seminatural anthrome weekly cycle is clearest at a similar time of the year (mid-September to early October) and has a partially overlapping geographical distribution (Southern hemisphere Africa and South America, Fig. 13). During this period, fire activity in Croplands during this strongly cyclic period displays a clear Sunday minimum and is concentrated in regions that are predominantly Christian and/or where labor regulations concerning a weekly period of rest are enforced^23,26^. Evidence of a weekly cycle is weaker in the Seminatural anthrome and not so much as a Sunday minimum, but more as a weekend effect (Table 5). In Rangelands, the time of the year when the weekly cycle in vegetation burning is most evident goes from late December to early February. Fire activity is concentrated in the tropical savannas of the Northern hemisphere, mainly in Muslim sub-Saharan Africa (see Fig. 14). Unlike at other times of the year, fire counts on Thursday and Friday, the religious and civil days of rest in the region, are inside the credible band for Sunday fire counts, suggesting the influence of religious practices on the weekly fire cycle^23^.

## Discussion and Conclusions

The present study revisited the topic of global weekly fire cycles, previously addressed by^26^ and^27^; We used original MCD14ML data, without any form of gridding or normalization of fire counts that appear to have caused the problems described in the Erratum of^26^. These problems were not fully corrected, as shown in the Supplementary Information Table 1, and cast doubt upon the reliability of their results. Our geographical stratification of the analysis was defined a priori, avoiding potential biases introduced by post-hoc spatial domain selection and relies on an explicit hypothesis that the likelihood and features of active fire weekly cycles are anthrome-specific. The anthrome-based spatial stratification provides insights into the influence of land use intensity on the occurrence of fire weekly cycles.

Employing statistical methodologies more appropriate for the detection of weekly cycles in time series of active fire counts, and for determining the weekday with the fewest fires, we reliably detected a weekly global fire activity cycle with a Sunday minimum, and Table 3 shows that for Saturday there is a reasonable probability that the corresponding mean function is inside the credible band for Sunday. Also, Figs 7 and 8 show that the estimated mean function for Sunday is almost everywhere below the estimated mean function for the weekdays and almost always very close or even below the lower confidence bounds of the week days. However, contrary to the other days of the week, the estimated mean function for Saturday is always inside the credible bands for Sunday. This finding, associated with the reasonable high probability 0.7019 displayed in Table 3, indicates that rather than a Sunday minimum, there actually is a weekend minimum in global active fire counts.

Our research expanded previous continental-scale work on weekly cycles of fire activity when the data are disaggregated by anthrome^23^, by identifying Croplands (40.19% of all fires) as the anthrome with the strongest fire weekly cycles, which reaffirms the importance of land management intensity as a driver of the cycles. Table 6 shows strong evidence that the Sunday mean function for Croplands is statistically smaller than the mean function for every other day of the week including Saturday, revealing a Sunday effect on the decrease of cropland fire activity, rather than the weekend effect observed at global scale. Fire weekly cycles are weaker in Rangelands and Seminatural anthromes, which jointly account for 53.46% of all fires than in Croplands, due to lower land management intensity and less strict control over fire size and duration. Our results showing that anthromes exhibiting weekly cycles in fire activity cover 70% of the global land surface are in line with the findings of^24^ and^41^, who considered that anthropogenic vegetation burning clearly dominates vegetation fires at the global scale, and that fires started by lightning are responsible for a minor proportion of global biomass burning. According to^24^, exceptions are Canada, Russia, and the USA, where an estimated 85%, 49.0% and 67.6% of the burned biomass, respectively, is due to lightning fires. Their results indicate an amount of biomass burning due to anthropogenic fires ranging from 3.5 to 3.9 billion tons of dry matter per year (Pg dm/yr). Most of this biomass burning occurs in the savannas of Sub-Saharan Africa that, in the terms of the present study, correspond to a mixture of cropland, rangeland and seminatural anthromes.

Figures 9 and 10 show that there are no weekly fire cycles in the anthromes that accounf for a small percentage of global active fires (6.35%), and that are the most (Settlements and Villages) and least transformed (Wildlands) by human action. In Wildlands there is a much larger proportion of lightning-caused ignitions than in the other anthromes, and fires often burn without any attempts at suppression. This more natural pattern of burning is not conducive to the emergence of weekly cycles. In Settlements and Villages, high population density and landscape fragmentation lead to limited use of fire and, by definition, small amplitude in the number of fires per weekday. In conclusion, there is a weekly cycle in global active fire data, mostly due to the use of fire in croplands and, to a lesser extent, in rangelands and seminatural areas. Sunday is the weekday with the fewest fires at the global scale, because labour laws in most countries and Christianity, the largest global religion, prescribe it as the weekday of rest^6^ and^33^ showed the influence of human activity on the seasonality of global vegetation burning and that kind of influence is now evident at the weekly time scale. Fire-enabled dynamic vegetation models with daily temporal resolution and chemical weather forecast models will benefit from incorporating representations of weekly cycles in fire activity.

## Supplementary information

Table 1

## Data Availability

All data used in our study are publicly available at https://earthdata.nasa.gov/c5-mcd14dl (active fires) and http://ecotope.org/anthromes/v2/data/ (anthromes map, version 2). The code to run the model in R-INLA can be obtained from the authors upon request.
