# Supplementary material for: Anthromes displaying evidence of weekly cycles in active fire data cover 70% of the global land surface
Source: Sci Rep. 2019 Aug 6;9:11424. doi: 10.1038/s41598-019-47678-4 (PMC6684812; doi:10.1038/s41598-019-47678-4)
Supplement: Supplementary file 1 — Table 1 [file 41598_2019_47678_MOESM1_ESM.docx]

**Anthromes displaying evidence of weekly cycles in active fire data cover 70% of the global land surface**

J. M. C. Pereira, M. A. Amaral Turkman, K. F. Turkman and D. Oom

Table 1. Comparison of Earl et al. (2015) and Earl and Simmonds (2018) MODIS active fire counts with those from other sources.

|  | **Earl et al. (2015)** | **Earl & Simmonds (2018)** | **Oom & Pereira (2013)** | **Csiszar et al. (2005)** | **Korontzi et al. (2006)** | **Lin et al. (2012)** | **This study** |
| --- | --- | --- | --- | --- | --- | --- | --- |
| Total  Annual mean  Range 2003 – 2009  Range 2001 – 2013  Range 2001 – 2016 | 83 574 828^1^  6 428 833^1^  ≈ 5 500 000^3^ -  ≈ 7 000 000^3^  ≈ 5 500 000^3^ to  ≈ 7 900 000^3^ | ≈ 40 000 000^7,9^  2 500 000^9^  ≈ 2 250 000^7^ -  ≈ 2 600 000^7^  ≈ 2 100 000^7^ -  ≈ 2 800 000^7^    ≈ 2 150 000^7^ -  ≈ 2 800 000^7^ | 36 191 511^2^  4 021 279^2^  4 061 749 -  4 670 379 |  |  |  | 43 416 629^8^  4 341 663^8^ |
| 2001 | ≈ 8 000 000^3^ | ≈ 2 800 000^7^ | 1 597 241^4^ | 1 254 090 | 1 577 952 |  | 1 597 241^4^ |
| 2002 | ≈ 7 500 000^3^ | ≈ 2 750 000^7^ | 3 349 321^5^ | 1 225 165 | 1 572 884 |  | 3 349 321^5^ |
| 2003-2006 mean | ≈ 6 500 000^3^ | ≈ 2 500 000^7^ | 4 533 376 |  |  | 4 509 451 | 4 533 376 |
| Data source | TERRA + AQUA collection 5  MOD14A1^6^, gridded at 0.1° | TERRA+AQUA collection 6  MOD14A1^6^, gridded at 0.1° | TERRA+AQUA collection 5, MCD14ML | TERRA collection 4, daytime only | TERRA collection 4 | TERRA+AQUA  collection 5, MCD14ML | TERRA+AQUA collection 5, MCD14ML |

Table 1. Comparison of Earl et al. (2015) and Earl and Simmonds (2018) MODIS active fire counts with those from other sources.

^1^2001-2013

^2^2001-2009

^3^Estimated visually from figure 2f

^4^Only TERRA data were available in 2001

^5^AQUA data available starting in July

^6^NASA Earth Observations website (<http://neo.sci.gsfc.nasa.gov/view.php?datasetId=MOD14A1_M_FIRE>)

^7^Estimated visually from figure 2.

^8^July 2002 – July 2012

^9^2001-2016
